# Brinkmanship in intragenomic conflict

**DOI:** 10.1098/rspb.2025.0488

**Published:** 2025-04-23

**Authors:** Patrick Kennedy, Andrew D. Higginson

**Affiliations:** ^1^ School of Biological Sciences, University of Bristol, 24 Tyndall Avenue, Bristol BS8 1TQ, UK; ^2^ Centre for Research in Animal Behaviour, College of Life and Environmental Sciences, University of Exeter, Exeter, Devon EX4 4QG, UK

**Keywords:** intragenomic conflict, genomic imprinting, deterrence, war of nerves, developmental disorders, inclusive fitness, brinkmanship

## Abstract

When the Darwinian interests of genes in the genome collide, intragenomic conflicts evolve. Recent advances in social evolution predict that intragenomic conflicts shape diverse phenotypes. However, principles governing which side wins remain unresolved. Here, we use game theory to predict that power asymmetries arise from differences in appetite for risk between rival genes in ‘wars of nerve’. We focus on ‘genomic imprinting’: differing expression between alleles inherited from mothers and fathers. Escalating conflict is commonly believed to risk damaging the whole organism. We show that genes can exploit risk strategically: genes prepared to take greater risks with the body’s vulnerability to disorders and mortality gain coercive advantages, deterring countermoves. Kin selection generates differences in appetite for risk: for instance, if harm to the body frees resources for maternal siblings, genes from mothers have less to lose from gambling with the current body than do genes from fathers. Seemingly maladaptive developmental risks can be adaptively useful for higher-nerve genes, much as political states manipulate risk to coerce rivals. Our results suggest a determinant of power alongside the ‘loudest voice prevails’ principle, and call for empirical investigation of the extent and means by which risks of imprinting-related disorders are amplified by intragenomic brinkmanship.

## 1. Introduction

*‘We’re eyeball to eyeball, and I think the other fellow just blinked.’*

*—Dean Rusk, US Secretary of State, 1962*

In sexual genomes, genes can be rivals [1]. Despite temporarily sharing the same body, genes inherited from mothers (‘matrigenes’) and genes inherited from fathers (‘patrigenes’) can disagree over the phenotype that would best serve their competing interests [1–7], since each may be differently related to other organisms affected by their body. Natural selection favours each side pulling the body towards its preferred phenotype, fuelling an evolutionary arms race. When matrigenes and patrigenes differ in gene expression, the locus is said to show ‘genomic imprinting’ [8,9]. Consequently, intragenomic conflict is the leading explanation for the substantial numbers of imprinted loci detected in many animal genomes [10,11]. Imprinting-based conflicts have now been predicted in a wide diversity of traits, including the evolution of pregnancy [12,13], dispersal [14], sex allocation [15], intergroup conflict [16], menopause [17], plant seed size [18], social cognition [19], prey defences [20], haplodiploidy [21], language [22], sleep [23] and eusociality [24,25]. In each case, unless there are mechanistic constraints on adaptation, the resulting conflict is thought to stop only when it interferes intolerably with other phenotypic traits [12,26]. Pleiotropy, for instance, is thought to create a brink scenario: by escalating a conflict too far, an allele risks destabilizing gene networks, impairing the body’s survival and reproduction [26,27].

Principles for which side ultimately prevails in intragenomic conflicts have remained unresolved. We lack a general theory of power asymmetries, and ‘genetic power’ [28] has been suggested to be ‘intrinsically unpredictable’ [4, p. 113]. This lack of theory constrains our ability to predict whether any given phenotype should be closer to the matrigenic or patrigenic preference. The only general rule is the ‘loudest voice prevails’ principle [29]: within a single locus, the allele that favours a higher level of total expression is continually counteracted over evolutionary time by the rival that favours a lower level of total expression, with continuing adjustments in opposing directions until the allele with the lower preference evolves to silence itself completely; at this point, it cannot reduce expression at the locus any further. The field is left open for the allele with the higher preference to continue evolving to a higher expression level until it reaches its preferred value for the locus as a whole. However, the ‘loudest voice prevails’ principle does not predict which side will ultimately prevail in shaping the phenotype, because it applies only to expression levels at a single locus. Phenotypes are controlled by multiple loci [30], including enhancers, inhibitors and modifiers.

Here, we propose that genetic power—and hence the outcome of intragenomic conflict—is determined by a war of nerves. Escalation in intragenomic conflict is widely held to risk harming the wider organism [26]. In a game of competitive risk-taking, we argue that matrigenes and patrigenes will frequently differ in the extent to which they are prepared to hold their nerve (‘brinkmanship’), and hence the extent to which each will deter countermoves by rivals.

At least three reasons have been suggested for why escalating intragenomic conflict should raise the risk of mutual disaster. First, several authors have highlighted the collateral risks produced by pleiotropy [26,27]: as conflict over a disputed phenotype fuels increased gene expression, the risk that undisputed phenotypes will be dragged from their optimum increases. Second, Frank & Crespi [31] argue that, in a tug-of-war, two parties pulling strongly in opposite directions become dangerously vulnerable to perturbations: if one side is inadvertently dislodged by accidental loss of imprinting, the phenotype may lurch so far towards the other side that it may overshoot that side’s optimum, the developmental equivalent of a contestant slipping in a tug-of-war and the other falling backwards. Frank & Crespi [31] suggest that various cancers and psychiatric disorders may be linked to such chance perturbations of a ‘precarious balance’ between rival genes produced by escalated conflict. Third, Wilkins [32] and Wilkins & Úbeda [26] suggest a process of ‘conflict-induced decanalization’: when mean gene expression at a focal locus increases, stochasticity in gene expression often increases substantially, raising the risk of unusual and pathological outcomes. These three hypotheses—pleiotropy, ‘precarious balance’ and conflict-induced decanalization—all predict greater risk to the organism’s survival or reproductive success with greater escalation in intragenomic conflict.

To illustrate the potential for brinkmanship to shape the outcome of intragenomic conflict in general, we explore a classic case of conflict: mammalian pregnancy [12,13]. In a promiscuous society, patrigenes in a fetus have little expectation of also being in maternal siblings, and so are indifferent to sibling fitness. A patrigene in a fetus would maximize its Darwinian fitness if the fetus were to monopolize resources at the expense of future siblings. In contrast, fetal matrigenes disagree: since they have a 50% chance of also being transmitted to any future sibling, they have a kin-selected interest in a sibling’s success, and so prefer that the fetus show self-restraint rather than greed. An internal struggle occurs in the fetus, as patrigenes and matrigenes attempt to influence pathways involved in fetal resource consumption.

Each act of escalation may bring the organism closer to the brink of harm. Viewed as a war of nerves, matrigenes and patrigenes in a mammalian fetus may have either more to gain or less to lose from pulling harder (escalating risk) in the tug-of-war over the phenotype. For instance, the fetus may be more expendable for a matrigene: if the fetus fails via early spontaneous abortion or becomes an uncompetitive offspring, unspent resources may be recouped by siblings. Such a matrigene may have less to lose from going to the brink. Alternatively, patrigenes might have the greater appetite for risk, as they may have more to gain: a patrigene has only a single opportunity for success in this womb, while matrigenes may be more indifferent about the division of resources and hence prepared to concede early. To distinguish between verbal possibilities and identify how power is determined in biological games of brinkmanship, a formal model is needed.

Thomas Schelling, game theorist of the Cold War, illustrated the logic of brinkmanship with a famous metaphor [33]. Two mountaineers are arguing on a ledge, squabbling over dwindling rations. They are strapped together: if one slips, both will fall. Any step towards the precipice raises the risk of mutual disaster. By stepping further towards the uncertain brink, each climber hopes to escalate risk until the other relents. Schelling’s climbers are found in diverse real games of negotiation, from the Cuban missile crisis [34] to political clashes, business deals and disputes between parents and children [35], which are understood using game theory. Here, we assume that alleles in sexual genomes also find themselves in games of competitive risk-taking, and thus use evolutionary game theory to predict outcomes.

## 2. Model and results

### (a) Description of the game

To address whether differences in risk aversion between alleles should shape the outcome of intragenomic conflict, we use a coevolutionary model of escalation between patrigenes and matrigenes. We consider two rival actors in a tug-of-war: all the matrigenes versus all the patrigenes, which we treat as separate agents. Each side must choose how much to tug, producing more of gene products that pull the shared body’s phenotype towards its own optimum and frustrating gene products produced by the rival alleles. However, each act of escalation may carry the body closer to the brink of intolerable damage.

We consider the standard scenario for imprinting-related conflict in mammals [13], involving two siblings: a ‘first’ and a ‘second’ offspring. The first offspring is currently developing, and the second offspring is an upcoming offspring of the same mother. The dispute occurs between patrigenes and matrigenes within the first offspring, over how much of the mother’s finite resource to consume (henceforth, ‘greediness’). By claiming more resources, a first offspring reduces the resources available for the second offspring. Because first and second offspring may have different fathers, matrigenes and patrigenes in the first offspring have different expected relatedness to alleles in the second offspring, and so differ in their preferences for how greedy the first offspring should be.

Within the first offspring, let p denotes escalation by patrigenes, m denotes escalation by matrigenes and $k=\frac{p+m}{2}$ denotes their average escalation (measuring divergence in expression). We assume that each act of escalation increases the risk that undisputed aspects of the body’s phenotype are affected. We allow for this risk to arise through either a steady accumulation of damage to the body, such that it has lower fitness, or through an all-or-nothing developmental catastrophe that occurs with some probability; in either case, conflict escalation impairs the first offspring’s expected survival or future reproductive success. We assume that the expected cost to fitness is an accelerating function of k:


(2.1) $$\begin{array}{c} h\left(k\right)=\lambda k^{2}, \end{array}$$

where λ is a constant that determines the level of ‘riskiness’ associated with escalation.

Both first and second offspring have baseline fitness α. The expected fitness of the first offspring is:


(2.2) $$\begin{array}{c} W_{1}=\alpha +f\left(y\right)-h\left(k\right), \end{array}$$

where the subscript ‘1’ denotes the first offspring. The variable y is the proportion of a disputed resource it obtains, with 1-y going to the second offspring. The function fdetermines the fitness accrued from gaining a given share of the disputed resource. We assume that resource acquisition affects a body fitness with diminishing returns:


(2.3) $$\begin{array}{c} f\left(y\right)=1-{\left(1-y\right)}^{2}. \end{array}$$

The tug-of-war occurs in the first offspring. We use uppercase to denote trait values in that tug-of-war from the perspective of alleles in the second offspring. Hence, Y is the division of resources imposed upon these alleles as the outcome of the tug-of-war in their sibling’s body, while K is the average escalation that took place in that sibling body. Accordingly, the expected fitness of the second offspring is:


(2.4) $$\begin{array}{c} W_{2}=\alpha +f\left(1-Y\right)+h\left(K\right)q, \end{array}$$

where q is the fitness effect experienced by the second sibling if the first offspring suffers a loss of fitness. For instance, if a fatal developmental catastrophe befalls the first offspring, q is the proportion of resources that would have been conferred on the first offspring that can instead be recouped by the second offspring. Throughout, we refer to q as ‘resource recovery’.

We assume a large population of outbreeding diploids; a matrigene has a 50% chance of also being transmitted to its body’s maternal sibling, so a matrigene’s relatedness to the sibling body is always $r_{M}=\frac{1}{2}$. However, we allow a patrigene’s relatedness to the sibling body ($r_{P}$) to take any value from 0 (i.e. a fully promiscuous population) to $\frac{1}{2}$ (i.e., a fully monogamous population).

To evaluate selection on traits of interest, we apply the Taylor–Frank neighbour-modulated method [36] (electronic supplementary material, appendix A), which asks how the fitness of a focal agent is affected both by its own behaviour and by the behaviour of neighbours. As first and second offspring are equally common in the population, an allele has an equal chance of finding itself in each (so its expected fitness is the average over the two possibilities ($W=\frac{W_{1}+W_{2}}{2}$). If it is in a first offspring, the only way for the trait of interest to affect its fitness is through its own expression of that trait. We denote this ‘focal’ expression by lowercase p and m. If it is in a second offspring, the only way for the trait of interest to affect its fitness is through the expression of the trait by neighbouring alleles in the first offspring. We denote this ‘neighbour’ expression by uppercase P and M. We denote population-average values with overbars ($\overset{-}{m}$ and $\overset{-}{p}$). We assume that the two offspring are produced in quick succession, and that first and second offspring in the population then compete in the same pool for recruitment as next year’s adults; consequently, our first and second offspring cohorts do not differ in class reproductive value. Accordingly, selection favours a small increase in escalation by patrigenes ($\Delta \overset{-}{p}>0$) if:


(2.5) $$\begin{array}{c} \frac{\partial W_{1}}{\partial p}+\frac{\partial W_{2}}{\partial P}r_{P}>0 \end{array}$$

and a small increase in escalation by matrigenes ($\Delta \overset{-}{m}>0$) if:


(2.6) $$\begin{array}{c} \frac{\partial W_{1}}{\partial m}+\frac{\partial W_{2}}{\partial M}\frac{1}{2}>0, \end{array}$$

where all traits are evaluated at their population-average values.

The two sides in a tug-of-war aim to pull the outcome towards their ideal preference. In the absence of escalation, the optimum division of resources from the perspective of each side is $\frac{1}{1+r}$ (electronic supplementary material, appendix B), where r is the relatedness to the second offspring (i.e. $r=r_{M}$ for matrigenes and $r=r_{P}$ for patrigenes). For matrigenes ($r_{M}=\frac{1}{2}$), the optimum is therefore $O_{M}^{*}=\frac{2}{3}$. For patrigenes the optimum is $O_{P}^{*}=\frac{1}{1+r_{P}}$. In a fully promiscuous population, patrigenes favour the first offspring completely monopolizing resources ($O_{P}^{*}=1$), since they are unrelated to the second offspring ($r_{P}=0$).

Let success in the tug-of-war depend on the difference in escalation efforts between the matrigenes and patrigenes, such that the outcome (x) is at the patrigene preference when x=1 and the matrigene preference when x=0 and at an intermediate value between these preferences when 0<x<1:


(2.7) $$\begin{array}{c} x=\frac{1}{2}+\frac{p-m}{2}. \end{array}$$

Here, the status quo involves a fair split between the two parties’ preferences ($x=\frac{1}{2}$), which occurs if neither side invests any effort in fighting (p=0 and m=0) or if their efforts are positive but equal (p=m). We allow for negative trait values p<0 and m<0 (pushing the phenotype away from one’s own optimum).

The resulting division of resources (the proportion y gained by the first offspring) after the tug-of-war within the first offspring’s genome is:


(2.8) $$\begin{array}{c} y=O_{P}^{*}x+\frac{2}{3}\left(1-x\right). \end{array}$$

The remaining proportion (1-y) is acquired by the second offspring.

Accordingly, the model makes a distinction between the phenotype ultimately shown by the organism and the underlying tug-of-war. The organism’s phenotype is y. Matrigenes and patrigenes differ in their preference for what y should be. These two preferences ($O_{M}^{*}$ and $O_{P}^{*}$) form the ‘borders’ of the tug-of-war: the values of y that each side would select if it had complete control over the phenotype and if there were no risk. The escalation levels (m and p) denote how much effort each side invests in the tug-of-war, and x indicates where the tug-of-war ends on the continuum between the preferences.

### (b) Power in a simultaneous game of escalation

**Figure 1.**
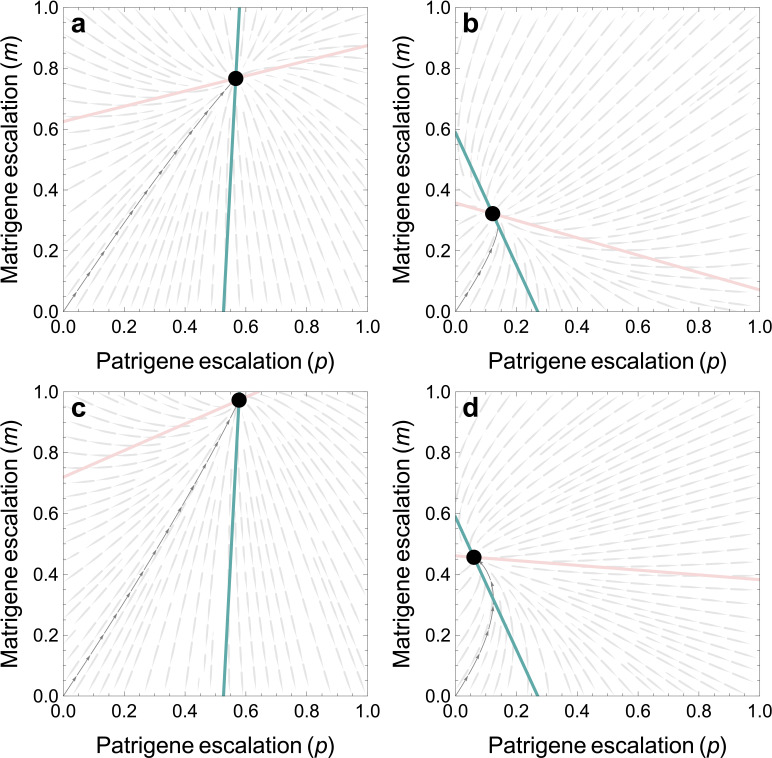
Best responses of patrigenes (blue) and matrigenes (light red). The joint ESS from coevolution between matrigenes and patrigenes is at the black circle. Coevolution from a starting point of zero escalation is shown in grey. (a,c) Lower riskiness (*λ* = 0.1); (b,d) higher riskiness (*λ* = 0.3); (a,b) no resource recovery (*q* = 0); and (c,d) resource recovery (*q* = 0.7). In (a–d), we assume a promiscuous society (*r*_P_ = 0).


We now solve for the best responses by each side (electronic supplementary material, appendix C). The candidate joint evolutionarily stable strategy (ESS) for coevolution in escalation (black circle in figure 1) occurs at:


(2.9) $$\begin{array}{c} p_{S}^{*}=\frac{\left(1-2r_{P}\right)\left(4r_{P}-2+3\left(1+q\right)\left(1+r_{P}\right)\lambda \right)}{6\left(1+r_{P}\right)\left(q-5-2r_{P}+4qr_{P}\right)\lambda }, \end{array}$$

(2.10) $$\begin{array}{c} m_{S}^{*}=\frac{\left(1-2r_{P}\right)\left(4r_{P}-2-3\left(1+q\right)\left(1+r_{P}\right)\lambda \right)}{6\left(1+r_{P}\right)\left(q-5-2r_{P}+4qr_{P}\right)\lambda }, \end{array}$$

where the subscript ‘S’ denotes a game of simultaneous moves during development by patrigenes and matrigenes. We substitute these coevolutionary ESS levels of escalation into the contest success function (CSF) equation (2.7) to find the equilibrium outcome $x_{S}^{*}$ of the tug-of-war:


(2.11) $$\begin{array}{c} x_{S}^{*}=\frac{\left(q-2\right)\left(1+r_{P}\right)}{q-5-2r_{P}+4qr_{P}}. \end{array}$$

Note that λ does not appear in equation (2.11). Accordingly, the *absolute* level of risk (λ) does not affect the phenotypic outcome of the tug-of-war, in terms of whether it ends closer to the patrigenes’ or matrigenes’ optimum: for risk to shape the outcome by generating a war of nerves, it only matters that escalation carries some risk (λ>0). The absolute level of risk does, however, affect the levels of escalation at equilibrium equations (2.12) and (2.13): if risk is low, the parties continue escalating until reaching the outcome $x_{S}^{*}$ (figure 1a,c). If risk is high, the parties reach the same outcome ($x_{S}^{*}$) after only small levels of escalation (figure 1b,d).

In the model presented here, matrigenes gain genetic power: they have higher nerve and hence are prepared to step closer to the brink (cf. electronic supplementary material, figure S1a and S1c). The difference in nerve arises from two advantages.

First, genes can differ in the amount they stand to gain from pulling the tug-of-war towards their optimum, and therefore in how much risk they are prepared to tolerate before conceding. A difference in fitness benefits for the two offspring arises due to diminishing returns to fitness from increasing resources: the more resources a body gains, the less valuable any additional unit becomes. In a fully promiscuous society, patrigenes are unrelated to the second offspring ($r_{P}=0$), so their interest in the division of the disputed resource arises only through the first offspring, where the total available fitness payoff is lower. In the pursuit of larger total benefits from both offspring, matrigenes are therefore prepared to accept greater costs.

Second, genes can differ in how much they stand to lose from risking the body they inhabit. Although neither side wants the body to be harmed or die, the inclusive fitness costs of harm are lower for matrigenes: when resource recovery q>0, the harm is partially recouped by releasing resources for kin. After the failure of a fetus or fledgling, unspent parental resources may be redirected at siblings. Paradoxically, therefore, suffering higher kin competition than patrigenes is a source of bargaining strength for matrigenes: it reduces the relative cost of disaster, allowing them to bargain closer to the brink. This effect amplifies the matrigenes’ advantage, although matrigenes typically still maintain higher nerve at q≈0, for the first reason. For instance, in a fully promiscuous society ($r_{P}=0$), the outcome of the tug-of-war equation (2.11) simplifies to $x_{S}^{*}=\frac{q-2}{q-5}$, and therefore varies from partial matrigene victory ($x_{S}^{*}=0.4$) when there is no resource recovery by second siblings (q=0) to high matrigene victory ($x_{S}^{*}=0.25$) when there is full resource recovery by second siblings (q=1).

When risk (λ) is high relative to the potential gains from winning the tug-of-war, it can pay for one party to abandon the tug-of-war entirely. Here, patrigenes adopt a negative strategy ($p_{S}^{*}<0$) (electronic supplementary material, figure S2b–d). This involves actively pushing the outcome away from their own preference and towards the matrigenes’ optimum: reducing the risks associated with conflict becomes more desirable than winning the struggle over the phenotype.

### (c) Patrigenes as first movers

Above, we considered a game of simultaneous moves by different genes in the genome, and found that the side with higher nerve prevails. Next, we turn to a game of sequential moves. Phenotypes are the outcomes of complex pathways, often with multiple enhancer and inhibitory controls and nonlinear interactions. Windows for more easily enhancing or inhibiting regulatory pathways and gene networks may change as development progresses. When a pathway can be more easily pushed towards a particular phenotypic outcome at a particular time, the party that prefers that outcome enjoys a window in which they have greater leverage to act than their rival. Here, we consider whether allowing for sequential moves changes the strategies and outcomes in the tug-of-war.

Intuitively, opportunities to act first during development may confer a strategic advantage. When the players act simultaneously, they both escalate risk together—and both stop when their combined escalation becomes intolerable. However, if one side can move first, they may be able to secure a greater share of the outcome by manipulating risk: e.g. by raising risk to a level that is intolerable for patrigenes, matrigenes may deter patrigenes from entering the tug-of-war. The limited budget for relatively safe escalation would already have been exploited by matrigenes, and patrigenes would decline to escalate further. We adjusted the model to test this verbal argument.

First, consider a game in which patrigenes have the first move. At a subgame-perfect equilibrium, patrigenes act as if knowing that matrigenes will respond with their best response to whichever escalation level they choose. The best-response function for matrigenes follows that in the simultaneous game (electronic supplementary material, equation C5, appendix C), with a slight difference. Since matrigenes are now moving second, they must respond to the behaviour of the specific patrigenes in the body they inhabit (p), rather than to the population average value $\overset{-}{p}$. Accordingly, we replace $\overset{-}{p}$ with p (electronic supplementary material, equation C5) to provide the matrigenic best response in a game in which patrigenes move first (game order denoted by the subscript ‘PM’):


(2.12) $$\begin{array}{c} m_{PM}^{*}=\frac{3p\left(2-q\right){\left(1+r_{P}\right)}^{2}\lambda -\left(1+p\right){\left(1-2r_{P}\right)}^{2}}{3\left(q-2\right){\left(1+r_{P}\right)}^{2}\lambda -{\left(1-2r_{P}\right)}^{2}} \end{array}.$$

As above, to evaluate selection on a trait of interest using the Taylor-Frank method, we consider its effects on recipient genes, who can be in either the first or second offspring. When a focal recipient is in the second offspring body, the matrigene behaviour that matters is in the neighbour (first offspring) body. Accordingly, we set the neighbour matrigene trait value (M) to $M_{PM}^{*}$, which is identical to equation (2.12) but with P (patrigene behaviour in the neighbour body) in place of p. In the equations for fitness ($W_{1}$ and $W_{2}$), we then set $m=m_{PM}^{*}$ and $M=M_{PM}^{*}$ (electronic supplementary material, appendix D).

Selection favours a small increase in patrigene escalation when $\frac{\partial W_{1}}{\partial p}+\frac{\partial W_{2}}{\partial P}r_{P}>0$. Evaluating at $p=P=\overset{-}{p}$, setting change due to selection to zero, and rearranging for $\overset{-}{p}$ gives the optimum patrigene strategy $p_{PM}^{*}$:


(2.13) $$\begin{array}{c} p_{PM}^{*}=\frac{{\left(1-2r_{P}\right)}^{2}\left(1+q\left(r_{P}-2\right)+4r_{P}\right)+3{\left(q-2\right)}^{2}{\left(1+r_{P}\right)}^{3}\lambda }{6{\left(q-2\right)}^{2}{\left(1+r_{P}\right)}^{3}\lambda -6{\left(1-2r_{P}\right)}^{2}\left(qr_{P}-1\right)}. \end{array}$$

Given that patrigenes are playing $p_{PM}^{*}$, we find the expected behaviour of the matrigenes at equilibrium by letting $\overset{-}{p}=p_{PM}^{*}$ in the matrigenes’ best-response function $m^{*}$ (electronic supplementary material, appendix C):


(2.14) $$\begin{array}{c} m_{PM}^{*}=\frac{{\left(1-2r_{P}\right)}^{2}\left(7-q\left(5r_{P}+2\right)+4r_{P}\right)-3{\left(q-2\right)}^{2}{\left(1+r_{P}\right)}^{3}\lambda }{6{\left(q-2\right)}^{2}{\left(1+r_{P}\right)}^{3}\lambda -6{\left(1-2r_{P}\right)}^{2}\left(qr_{P}-1\right)}. \end{array}$$

To find the expected outcome of the tug-of-war in this sequential game ($x_{PM}^{*}$), we substitute $p_{PM}^{*}$ and $m_{PM}^{*}$ into the CSF equation (2.7):


(2.15) $$\begin{array}{c} x_{PM}^{*}=\frac{1}{2}+\frac{p_{PM}^{*}-m_{PM}^{*}}{2}=\frac{{\left(q-2\right)}^{2}{\left(1+r_{P}\right)}^{3}\lambda }{{\left(1-2r_{P}\right)}^{2}\left(1-qr_{P}\right)+{\left(q-2\right)}^{2}{\left(1+r_{P}\right)}^{3}\lambda }. \end{array}$$

In the baseline scenario where second offspring in a promiscuous society ($r_{P}=0$) are unable to recover resources when catastrophe occurs to the first offspring (q=0), $x_{PM}^{*}$ simplifies to:


(2.16) $$\begin{array}{c} x_{PM}^{*}=\frac{4\lambda }{1+4\lambda }. \end{array}$$

Since λ is present in equation (2.16), whether first-movers gain power over the resulting phenotype depends on the level of risk. Substituting the solutions for $x_{S}^{*}$ (equation (2.11)) and $x_{PM}^{*}$ (equation (2.16)) into the inequality $x_{PM}^{*}>x_{S}^{*}$, and rearranging for λ, gives the critical level of risk $\lambda_{PM}^{∙}$ above which patrigenes gain increased power over the resulting phenotype compared to the simultaneous game:


(2.17) $$\begin{array}{c} \lambda_{PM}^{∙}=\frac{{\left(1-2r_{P}\right)}^{2}}{3\left(2-q\right){\left(1+r_{P}\right)}^{2}}. \end{array}$$

**Figure 2.**
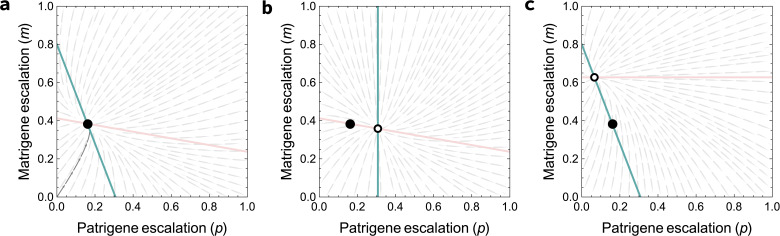
Deterrence requires negative best responses to escalation. (a) In the simultaneous game, the equilibrium occurs when each side plays its best response to the other. Matrigene and patrigene best responses to the other side are shown in red and blue, respectively. (b) When patrigenes move first, knowing that matrigenes will respond with their best response, they can deter matrigene escalation if the matrigene best response (red) has a negative slope in relation to rising patrigene escalation, moving the outcome from the simultaneous game equilibrium (black dot) to the new equilibrium (empty dot). (c) Similarly, when matrigenes move first, they can deter patrigene escalation if the patrigene best response (blue) has a negative slope in relation to rising matrigene escalation, moving the outcome from the simultaneous game equilibrium (black dot) the new equilibrium (empty dot). Panels plotted at *λ* = 0.25, *q* = 0.1.

When the risk is low ($\lambda <\lambda_{PM}^{∙}$), having to move first compromises the patrigenes’ power: the outcome ($x_{PM}^{*}$) is closer to the matrigene optimum (x=0) than in the simultaneous game ($x_{S}^{*}$). Matrigenes credibly threaten to meet patrigene escalation with further escalation, which has a deterrent effect on the first-moving patrigenes. Matrigene willingness to retaliate shown in figure 1: when $\lambda <\lambda_{PM}^{∙}$, the slope of the matrigene best response is positive (figure 1a*,*c). In contrast, when risk is higher than the critical level ($\lambda >\lambda_{PM}^{∙}$), escalation by first-moving patrigenes reduces the incentive for additional escalation by matrigenes, by consuming more of the remaining budget of risk that matrigenes are prepared to tolerate. Accordingly, matrigenes will respond to patrigene escalation by reducing their escalation compared to the simultaneous game. This deterrence effect of patrigene escalation on matrigene escalation shown in figure 1: when $\lambda >\lambda_{PM}^{∙}$, the slope of the matrigene best response is negative (figure 1b,d). Intuitively, brinkmanship by first movers is only rational if it deters second movers, and deterrence requires that second movers have a negative best-response curve to your escalation (figure 2).

**Figure 3.**
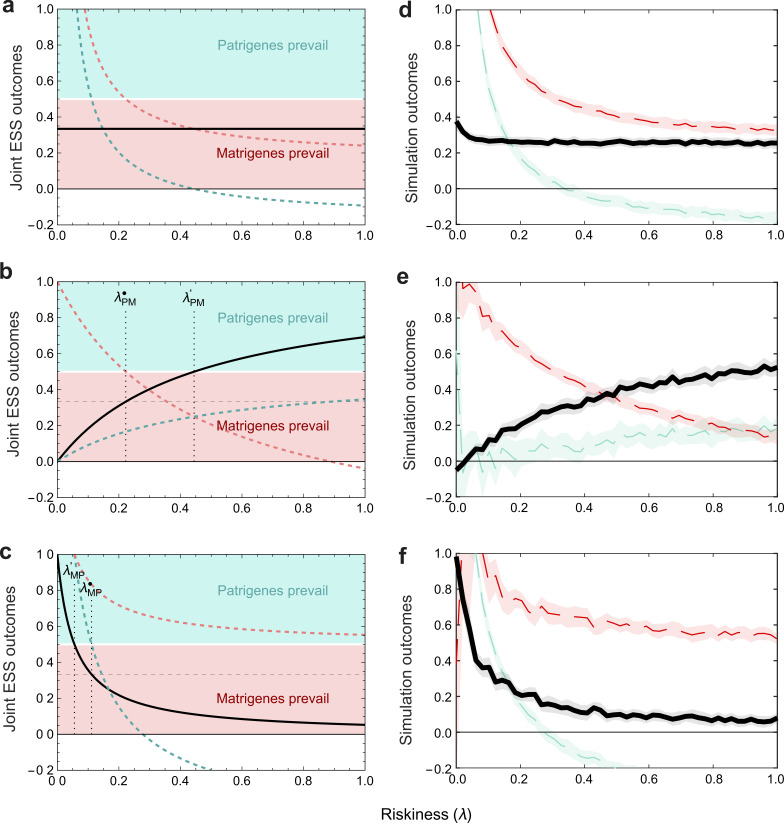
The phenotypic outcome of the tug-of-war is unaffected by total riskiness in the simultaneous game, but increasing riskiness gives power over the phenotype to the first-mover in sequential games. (a–c) Analytical solutions. (d–f) Individual-based simulations corresponding to (a–c). The black line shows the outcome of the tug-of-war (x*). When x* is below 0.5 (red region), the tug-of-war ends closer to the matrigene preference; when x* is above 0.5 (blue region), the tug-of-war ends closer to the patrigene preference. Matrigene and patrigene escalation levels at the joint ESS are shown by red and blue dashed lines, respectively. (a) Simultaneous game. (b) Sequential game with patrigenes moving first. (c) Sequential game with matrigenes moving first. In (b,c), the coevolutionary outcome in the simultaneous game (*x*_S_*) is shown by the horizontal dashed line. Increased riskiness offers first-movers a strategic opportunity to coerce second-movers, and therefore to obtain a bargaining strength. In (b) patrigenes do better than in the simultaneous game (*x*_S_*) when *λ* > *λ*_PM_^•^, and prevail over matrigenes (*x*_PM_* > 1/2) when *λ* > *λ*_PM_'. In (c) matrigenes do better than in the simultaneous game (*x*_S_*) when *λ* > *λ*_MP_^•^, and prevail over patrigenes when *λ* > *λ*_MP_'. Plotted for *q* = 1/2 in a promiscuous society (*r*_*P*_ = 0). (d–f) Individual-based simulations corresponding to the analytical models in (a–c), showing trait values averaged over the period 100 000–250 000 generations: lines show mean trait values across 50 simulated values of λ, and we show standard deviations at each of these simulations.


In the simultaneous game, patrigenes were the losing party (figure 1). Here, however, when patrigenes can move first and riskiness is above $\lambda_{PM}^{,}$, the phenotype moves closer towards their preference (figure 3). In order to deter entry by matrigenes, they escalate further than they would have in the simultaneous game. When riskiness is above the additional threshold $\lambda_{PM}^{`}$, first-moving patrigenes gain a sufficiently strong advantage that they become the winning party in the tug-of-war (entering the blue region of figure 3b, where the outcome is closer to the patrigenes’ optimum than to the matrigenes’ optimum), even in promiscuous societies (electronic supplementary material, figure S2a–d). We find the threshold $\lambda_{PM}^{`}$ by setting $x_{PM}^{*}=\frac{1}{2}$ (the point at which patrigenes achieve equal success to matrigenes) and rearranging for λ:

(2.18) $$\begin{array}{c} \lambda_{PM}^{\prime }=\frac{1}{{\left(q-2\right)}^{2}}. \end{array}$$

When riskiness is above this threshold ($\lambda >\lambda_{PM}^{\prime }$), matrigenes have a more severely limited budget of remaining risk that they will tolerate, so patrigenes can successfully deter matrigene escalation enough to prevail in the tug-of-war.

### (d) Matrigenes as first movers

Above, we considered a scenario in which greater opportunity to enhance the first offspring’s greediness phenotype occurs first during development, and so patrigenes (the party with typically weaker nerve in the simultaneous game) had the first move. Here, we consider the opposite scenario, in which greater opportunity to *reduce* greediness occurs first during development, so matrigenes (the party that shows the higher appetite for risk in the simultaneous game) have the first move. We follow the same method as for patrigenes moving first to get the optimal strategies.

At the subgame-perfect equilibrium, matrigenes can be sure that patrigenes will respond with a rational conditional response. We solve for the optimum matrigene strategy $m_{MP}^{*}$ (electronic supplementary material, appendix E):


(2.19) $$\begin{array}{c} m_{MP}^{*}=\frac{{\left(1-2r_{P}\right)}^{2}\left(4-2r_{P}+q-5qr_{P}\right)+27\left(1+r_{P}\right){\left(qr_{P}-1\right)}^{2}\lambda }{2\left(1+r_{P}\right)\left(\left(2-q\right){\left(1-2r_{P}\right)}^{2}+27{\left(qr_{P}-1\right)}^{2}\lambda \right)}. \end{array}$$

Given that matrigenes are playing $m_{MP}^{*}$, we find the expected value of the best response for patrigenes by letting $\overset{-}{m}=m_{MP}^{*}$ in the patrigenes’ best-response function $p^{*}$ (electronic supplementary material, equation C3, appendix C):


(2.20) $$\begin{array}{c} p_{MP}^{*}=\frac{{\left(1-2r_{P}\right)}^{2}\left(8+2r_{P}-q-7qr_{P}\right)+27\left(1+r_{P}\right){\left(qr_{P}-1\right)}^{2}\lambda }{2\left(1+r_{P}\right)\left(\left(2-q\right){\left(1-2r_{P}\right)}^{2}+27{\left(qr_{P}-1\right)}^{2}\lambda \right)}. \end{array}$$

The expected outcome of the tug-of-war when matrigenes have the first move ($x_{MP}^{*}$) is then:


(2.21) $$\begin{array}{c} x_{MP}^{*}=\frac{1}{2}+\frac{p_{MP}^{*}-m_{MP}^{*}}{2}=\frac{\left(2-q\right){\left(1-2r_{P}\right)}^{2}}{\left(2-q\right){\left(1-2r_{P}\right)}^{2}+27{\left(qr_{P}-1\right)}^{2}\lambda }, \end{array}$$

which simplifies to $x_{MP}^{*}=\frac{2-q}{2-q+27\lambda }$ when $r_{P}=0$.

As above, we ask whether moving first leads matrigenes to gain power over the phenotype compared to the simultaneous game ($x_{MP}^{*}<x_{S}^{*}$). Substituting $x_{MP}^{*}$ (equation (2.21)) and $x_{S}^{*}$ (equation (2.11)) into this inequality, and rearranging for λ, gives the critical riskiness threshold $\lambda_{MP}^{∙}$ above which the outcome phenotype moves closer to the matrigenes’ preference:


(2.22) $$\begin{array}{c} \lambda_{MP}^{∙}=\frac{{\left(2r_{P}-1\right)}^{2}}{9\left(1+r_{P}\right)\left(1-qr_{P}\right)}, \end{array}$$

which simplifies to $\lambda_{MP}^{∙}=\frac{1}{9}$ when $r_{P}=0$, such that resource recovery q has no effect in a fully promiscuous society.

The critical threshold $\lambda_{MP}^{∙}$ is low. Consequently, matrigenes gain increased power for most levels of riskiness (figure 3c), and therefore consolidate their winning position (electronic supplementary material, figure 2e–h).

### (e) Bounded strategies

In the preceding models, we have allowed patrigene and matrigene escalation to adopt any value. In principle, this includes negative values (de-escalating by pushing the trait away from the agent’s ideal preference). This flexibility follows from viewing escalation as the product of multiple loci interacting on each side, rather than as the products of single loci whose expression cannot be negative. For completeness, we also consider bounded games, in which m and p are not permitted to be greater than 1 or less than 0. Once riskiness λ rises above a threshold in a context where non-negative escalation is not possible, the inability of the weaker side to adopt de-escalatory strategies (p<0) can force the stronger side to reduce their escalation to avoid intolerable risk (electronic supplementary material, figure S3, appendices F–H). Consequently, high riskiness can now make outcomes more equitable by reducing the advantage of making the first move. If strategies are constrained to be under 1 (a mechanistic upper limit on escalation), equitable outcomes arise at low levels of riskiness λ (electronic supplementary material, figure S4) as both sides are prepared to escalate to the maximum extent allowed without generating sufficient combined risk to lead to deterrence (figure 3b,c).

### (f) Fitness outcomes

Finally, we consider who ‘wins’ in terms of absolute inclusive fitness payoffs. We let $\Delta W_{1}$ denote the change in the reproductive success of the first sibling as a result of the tug-of-war (relative to a status quo in which there is no conflict, such that $\overset{-}{m}=\overset{-}{p}=0$ and the phenotype is therefore a compromise between the two sides’ optima, $x=\frac{1}{2}$), and similarly for $\Delta W_{2}$:


(2.23) $$\begin{array}{c} \Delta W_{1}=\left(W_{1}|m=m^{*},p=p^{*}\right)-\left(W_{1}|m=p=0\right), \end{array}$$

(2.24) $$\begin{array}{c} \Delta W_{2}=\left(W_{2}|M=m^{*},P=p^{*}\right)-\left(W_{2}|M=P=0\right). \end{array}$$

**Figure 4.**
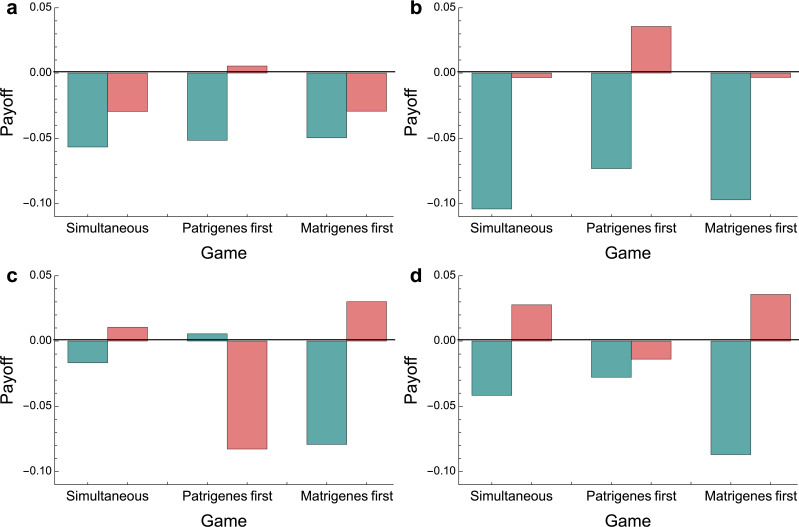
Absolute inclusive fitness payoffs at equilibrium. Payoffs to patrigenes are in blue; payoffs to matrigenes are in red. Panels are plotted at the same parameter values as in figure 2. (a) Low resource recovery (*q* = 0), low riskiness (*λ* = 0.1). (b) High resource recovery (*q* = 1), low riskiness (*λ* = 0.1), (c) low resource recovery (*q* = 0) and (d) high riskiness (*λ* = 1). High resource recovery (*q* = 1)*,* high riskiness (*λ* = 1). *r*_P_ = 0.

In absolute inclusive fitness payoffs ($\Delta W_{1}+r_{P}{\Delta W}_{2}$ for a patrigene, $\Delta W_{1}+\frac{1}{2}{\Delta W}_{2}$ for a matrigene), the necessity of having to fight in a tug-of-war is generally deleterious to fitness (figure 4): by increasing the risk to the organism, intragenomic conflict resembles a tragedy of the commons, in which individual self-interest leads to worse outcomes overall.

Nonetheless, absolute fitness gains through intragenomic conflict are possible relative to the status quo. For instance, when risk is low, second-moving matrigenes improve their position relative to the status quo (positive values for matrigene payoffs in the ‘patrigenes first’ game of figure 4a*,*b). As matrigenes credibly threaten to match patrigene escalation with further matrigene escalation, the patrigenes are deterred, and so matrigene fitness is increased (figure 4a,b) through greater success in the tug-of-war (levels of λ below $\lambda_{PM}^{`}$ in figure 3b). However, when risk is high, second-moving matrigenes do worse than in the status quo (figure 4c,d), as patrigenes can deter matrigenes from retaliation (levels of λ above $\lambda_{PM}^{`}$ in figure 3b), but matrigenes do better than the status quo in the sequential or simultaneous game. The only time patrigenes do better than the status quo is when they move first and resource recovery is low and riskiness is high (figure 3c).

## 3. Discussion

W. D. Hamilton famously described intragenomic conflict as ‘an eternal disquiet within’*,* a feud between ‘warring chromosomes … interlocked in strife’ [37, p. 135]. Here, we suggest that success in this war requires holding one’s nerve while raising risk: when there are risks from escalated conflict, alleles with less to lose from harming the organism—or more to gain from victory—gain bargaining strength, and hence have greater power over the organism’s phenotype.

A surprising implication of intragenomic brinkmanship is that some seemingly maladaptive failures of development, such as high abortion risk in very early-stage mammalian embryos or a greater incidence of cancer, may have an adaptive explanation. Prevailing in a war of nerves requires an actor to escalate risk to a level that is still tolerable for itself but intolerable for a rival. Accordingly, increasing some developmental vulnerabilities may be rational, either as a tolerable price of pulling in the tug-of-war (simultaneous model above) or as a strategic ploy (sequential models above), much as nation states may deliberately raise the risk of inadvertent mutual destruction as coercive diplomacy. In a game of simultaneous escalation, we find that a war of nerves is generated even if the absolute level of risk associated with intragenomic conflict is very low: low risks simply invite greater escalation, until the outcome is the same as it would have been if risk were intense.

The current model for how power is determined within the genome is the ‘loudest voice prevails’ principle [29]. In our model, power is determined instead through differences in nerve. The two perspectives can be reconciled as dealing with different scales of conflict. The ‘loudest voice prevails’ principle applies within a single locus: the allele that favours higher expression achieves its optimum, as the rival is pushed to silence itself completely, subsequently leaving the field open for the first allele to dictate the outcome. The war of nerves applies across loci: patrigenes and matrigenes as a whole form the two sides, and any particular imprinting conflict may involve multiple loci of small effect and modifiers. This aligns with the evidence: multiple imprinted loci often shape the resulting phenotype, forming an imprinted gene network [38,39].

The simultaneous game requires few assumptions: genes do not need to detect or use any information about the strategy of the specific rival gene in the same body’s genome. Instead, they simply evolve in response to the average behaviour of rivals in the population. However, in games where genes can move sequentially during development, we have assumed that second-moving alleles will ultimately evolve to use information about the behaviour of the first-moving allele in the same body (‘perfect information’). Conditional self-interested responses to the behaviour of other genes in the genome have not been tested empirically. However, a similar possibility has been raised before: Haig [40] suggests the possibility of ‘intrapersonal reciprocity’ (reciprocal social interactions between genes in the same genome), proposing relatively high levels of strategic flexibility that a gene might use to respond to its partner’s actions. For instance, patrigene behaviour might involve the retaliatory production of an inhibitor that only has an influence on developmental pathways if a pattern of matrigenic gene expression has already taken place. Similarly, to explain the observation that matrigenic and patrigenic expression across loci often appears to be correlated during development, Patten *et al.* [38] propose ‘regulatory eavesdropping’ (p. 4) between suites of matrigenes and patrigenes. Nonetheless, relaxing the assumption of perfect information to explore deterrence under uncertainty would be a useful extension of our model.

In the sequential games, we have also assumed that selection will reach the outcome that would have been reached if the alleles were rational inclusive-fitness-maximizing agents reasoning strategically about how their rivals will respond. Whether and when coevolutionary ESSs are ‘subgame-perfect’ has long been a topic of interest in evolutionary game theory and behavioural ecology. The classic answer is that it is expected under a ‘trembling hand’ process [41] or, similarly, when populations are large enough that mutants at different nodes in the game tree encounter each other sufficiently regularly [42]. However, if there are mechanistic or cost constraints preventing agents from evolving a fully flexible repertoire of best-response behaviours, simpler behavioural heuristics may be favoured by selection, and the outcome may not be subgame-perfect [43]. We have erred on the side of adaptationism: we have assumed that any such constraints are ultimately eroded in the long run. Models that explicitly examine the mechanistic constraints that matrigenes and patrigenes may face—and how they might use information within the cell—would be valuable.

Empirically, we do not yet know the shape of either the risk function or the CSF in any intragenomic conflict; both of these will play a key role in determining the extent and means of intragenomic brinkmanship. Indeed, a well-known feature of conflict studies is that different CSFs lead to different strategies [44]. In the present model, we have adopted simple risk and CSF functions, but a potentially fruitful avenue may be experimentally testing quantitative predictions for the shape of different mechanistically motivated risk functions associated with the three hypotheses (pleiotropy [26,27], ‘precarious balance’ [31] and conflict-induced decanalization [26]). Similarly, theoretical simulations may predict the most biologically plausible risk and CSF functions by modelling the effects of perturbations to imprinted gene networks.

Escalation is generally costly, generating a prisoners’ dilemma: if both parties could agree to self-restraint, both would do better (figure 4). In principle, mothers might escape the costs of intragenomic conflict in their offspring by signalling commitment to monogamy, either to fathers, who then pass the information to patrigenes in the gametes, or directly to offspring. If patrigenes can evolve to respond conditionally to credible signals of monogamy [45], monogamous mothers would escape two costs: (i) the outcome moves to the matrigenic optimum, which is closer to the maternal optimum; and (ii) any developmental risks associated with escalation are eliminated. The possibility that genomic imprinting may respond conditionally to cues and signals has attracted speculation: for instance, Haig [46] suggests that higher incidence of hypertension among pregnant women in short-term relationships [47] may be due to patrigenes increasing fetal resource consumption, given their reduced expected relatedness to future siblings. To our knowledge, however, the possibility that conditional imprinting may act as an adaptive driver of monogamy or monogamy signals has not been suggested. Species with intraspecific variation in monogamy, such as prairie voles [48], may provide opportunities for testing predictions of conditional imprinting.

Escalatory steps in intragenomic conflict may be diverse. The most likely form of escalation is simply the production of higher dosages of a gene product involved in either enhancing or inhibiting a gene pathway controlling a disputed phenotype. However, more direct acts of aggression can be envisioned. For instance, although tactical moves by alleles in intragenomic conflict remain little understood empirically, the evolution of trans-acting genes that tamper with the expression of rival imprinted alleles, including through the release of antisense RNA or through inhibitory interactions when homologous chromosomes are in contact during mitosis, offer a potential route to manipulation [11,38]. In some cases, loss of methylation may be linked to the activities of rival alleles [11]. Genes in parents may also attempt to tamper with offspring imprints [49]. Any hypothetical sabotage of undesirable imprints—such as removing or adding methyl groups on rival imprinting control regions (ICRs)—may substantially amplify risk, and form a potential arena for intragenomic brinkmanship besides the risks we discuss above. Dysregulation at ICRs is commonly associated with several imprinting-related disorders and oncogenesis, while recent data point to an association between unusual imprinting at a classic conflict locus (*IGF2*) and spontaneous abortion [50].

In this article, ‘risk’ refers to the danger of triggering a developmental catastrophe or eroding the body’s viability or fecundity. This use of the term ‘risk’ is different from a recent model of imprinting by Wilkins & Bhattacharya [51]. These authors present a model arguing that matrigenes and patrigenes may have different preferences for how much their shared body should pursue a life history with a lower variance in reproductive success (‘bet-hedging’). Our model does not involve or imply any conflicting preferences over bet-hedging traits (or indeed selection on the variance of reproductive success at all). However, although bet-hedging plays no role in our model, future models may explore whether bet-hedging can influence games of brinkmanship in general. For instance, if lower reproductive variance demands a cautious approach to risk-taking, a bet-hedging player (striving to reduce variance in reproductive success) might find itself disadvantaged in a war of nerves.

The literature on evolutionary arms races between predators and prey distinguishes several reasons for the emergence of power asymmetries. Dawkins & Krebs [52] introduced the ‘life–dinner principle’, which highlights why differences in stakes can drive prey to outperform predators: a gazelle is fighting for its life, but a cheetah is only fighting for its dinner. Scott & Queller [53] explore how mutation input affects outcomes. Of particular relevance to our model, Humphreys & Ruxton [54] have recently proposed a ‘dicey dinner principle’, in which prey can choose to adopt a behaviour that increases mutual risk as a ploy to induce a predator to abandon the chase, such as deliberately running into dangerous ground. Our model of intragenomic brinkmanship and Humphreys & Ruxton’s [54] model of dicey dinners both illustrate how asymmetrical power can arise through games of competitive risk-taking, and suggest that biologically general models of brinkmanship will be useful. Wars of nerve—with parties deliberately escalating risk as a deterrent—may occur widely in biology, from intragenomic conflict and predator–prey interactions to additional contexts such as struggles over resources and dominance within animal social groups. Within the genome, seemingly maladaptive failures of organismal development can be strategically useful credible threats wielded by genes in conflict.

## Data Availability

Simulation code used for the MATLAB model is included in the electronic supplementary material [55].

## References

[1] Gardner A, Úbeda F. 2017 The meaning of intragenomic conflict. Nat. Ecol. Evol. **1**, 1807–1815. (10.1038/s41559-017-0354-9) 29109471

[2] Gardner A. 2014 Genomic imprinting and the units of adaptation. Heredity **113**, 104–111. (10.1038/hdy.2013.128) 24496091 PMC4105447

[3] Gardner A, Welch JJ. 2011 A formal theory of the selfish gene. J. Evol. Biol. **24**, 1801–1813. (10.1111/j.1420-9101.2011.02310.x) 21605218

[4] Haig D. 2001 Genomic imprinting and kinship. New Brunswick, NJ: Rutgers University Press.

[5] Wilkins JF, Haig D. 2003 Inbreeding, maternal care and genomic imprinting. J. Theor. Biol. **221**, 559–564. (10.1006/jtbi.2003.3206) 12713940

[6] Burt A, Trivers R. 1998 Genetic conflicts in genomic imprinting. Proc. R. Soc. B **265**, 2393–2397. (10.1098/rspb.1998.0589) PMC1689541 10075542

[7] Babak T *et al*. 2015 Genetic conflict reflected in tissue-specific maps of genomic imprinting in human and mouse. Nat. Genet. **47**, 544–549. (10.1038/ng.3274) 25848752 PMC4414907

[8] Patten MM, Ross L, Curley JP, Queller DC, Bonduriansky R, Wolf JB. 2014 The evolution of genomic imprinting: theories, predictions and empirical tests. Heredity **113**, 119–128. (10.1038/hdy.2014.29) 24755983 PMC4105453

[9] Bartolomei MS, Ferguson-Smith AC. 2011 Mammalian genomic imprinting. Cold Spring Harb. Perspect. Biol. **3**, 1–17. (10.1101/cshperspect.a002592) PMC3119911 21576252

[10] Hu Y, Yuan S, Du X, Liu J, Zhou W, Wei F. 2023 Comparative analysis reveals epigenomic evolution related to species traits and genomic imprinting in mammals. Innov. **4**, 100434. (10.1016/j.xinn.2023.100434) PMC10196708 37215528

[11] Burt A, Trivers RL. 2006 Genes in conflict. Cambridge, MA: Harvard University Press. (10.4159/9780674029118)

[12] Haig D. 2015 Maternal–fetal conflict, genomic imprinting and mammalian vulnerabilities to cancer. Phil. Trans. R. Soc. B **370**, 20140178. (10.1098/rstb.2014.0178) 26056362 PMC4581023

[13] Haig D. 1993 Genetic conflicts in human pregnancy. Q. Rev. Biol. **68**, 495–532. (10.1086/418300) 8115596

[14] Farrell EJ, Úbeda F, Gardner A. 2015 Intragenomic conflict over dispersal. Am. Nat. **186**, E61–E71. (10.1086/682275) 26655360

[15] Wild G, West SA. 2009 Genomic imprinting and sex allocation. Am. Nat. **173**, E1–E14. (10.1086/593305) 19067608

[16] Micheletti AJC, Ruxton GD, Gardner A. 2017 Intrafamily and intragenomic conflicts in human warfare. Proc. R. Soc. B **284**, 20162699. (10.1098/rspb.2016.2699) PMC5326533 28228515

[17] Úbeda F, Ohtsuki H, Gardner A. 2014 Ecology drives intragenomic conflict over menopause. Ecol. Lett. **17**, 165–174. (10.1111/ele.12208) 24320989 PMC3912906

[18] Rodrigues JA, Zilberman D. 2015 Evolution and function of genomic imprinting in plants. Genes Dev. **29**, 2517–2531. (10.1101/gad.269902.115) 26680300 PMC4699382

[19] Isles AR, Davies W, Wilkinson LS. 2006 Genomic imprinting and the social brain. Phil. Trans. R. Soc. B **361**, 2229–2237. (10.1098/rstb.2006.1942) 17118935 PMC1764840

[20] Best R, Ruxton GD, Gardner A. 2018 Intragroup and intragenomic conflict over chemical defense against predators. Ecol. Evol. **8**, 1–8. (10.1002/ece3.3926) PMC5869269 29607027

[21] Normark BB. 2006 Perspective: maternal kin groups and the origins of asymmetric genetic systems—genomic imprinting, haplodiploidy, and parthenogenesis. Evolution **60**, 631. (10.1554/05-546.1) 16739447

[22] Hitchcock TJ, Paracchini S, Gardner A. 2019 Genomic imprinting as a window into human language evolution. BioEssays **41**, 1800212. (10.1002/bies.201800212) 31132171

[23] Faria GS, Varela SAM, Gardner A. 2019 The social evolution of sleep: sex differences, intragenomic conflicts and clinical pathologies. Proc. R. Soc. B **286**, 20182188. (10.1098/rspb.2018.2188) PMC6367171 30963856

[24] Haig D. 1992 Intragenomic conflict and the evolution of eusociality. J. Theor. Biol. **156**, 401–403. (10.1016/s0022-5193(05)80683-6) 1434666

[25] Dobata S, Tsuji K. 2012 Intragenomic conflict over queen determination favours genomic imprinting in eusocial Hymenoptera. Proc. R. Soc. B **279**, 2553–2560. (10.1098/rspb.2011.2673) PMC3350697 22378809

[26] Wilkins JF, Úbeda F. 2011 Diseases associated with genomic imprinting. In Progress in molecular biology and translational science (eds X Cheng, RM Blumenthal), pp. 401–445, vol. 101. (10.1016/B978-0-12-387685-0.00013-5) 21507360

[27] Rautiala P, Gardner A. 2023 The geometry of evolutionary conflict. Proc. R. Soc. B **290**, 20222423. (10.1098/rspb.2022.2423) PMC9904945 36750194

[28] Hurst LD, Atlan A, Bengtsson BO. 1996 Genetic conflicts. Q. Rev. Biol. **71**, 317–364. (10.1086/419442) 8828237

[29] Haig D. 1996 Placental hormones, genomic imprinting, and maternal–fetal communication. J. Evol. Biol. **9**, 357–380. (10.1046/j.1420-9101.1996.9030357.x)

[30] Czajkowski C, Karlin A, Sayle R, Milner-White EJ. 2001 Complex networks of interactions connect genes to phenotypes. Trends Biochem. Sci. **26**, 463–465. (10.1016/s0968-0004(01)01920-x) 11504611

[31] Frank SA, Crespi BJ. 2011 Pathology from evolutionary conflict, with a theory of X chromosome versus autosome conflict over sexually antagonistic traits. Proc. Natl Acad. Sci. USA **108**, 10886–10893. (10.1073/pnas.1100921108) 21690397 PMC3131809

[32] Wilkins JF. 2011 Genomic imprinting and conflict-induced decanalization. Evolution **65**, 537–553. (10.1111/j.1558-5646.2010.01147.x) 21029079

[33] Schelling T. 1966 Arms and influence. New Haven, CT: Yale University Press. (10.2307/j.ctt5vm52s)

[34] Sherwin MJ. 2020 Gambling with Armageddon: nuclear roulette from Hiroshima to the Cuban missile crisis. New York, NY: Alfred A. Knopf. (10.1111/pech.12461)

[35] Schelling T. 1960 The strategy of conflict. Cambridge, MA: Harvard University Press.

[36] Taylor PD, Frank SA. 1996 How to make a kin selection model. J. Theor. Biol. **180**, 27–37. (10.1006/jtbi.1996.0075) 8763356

[37] Hamilton W. 1996 Narrow roads of gene land, vol. 1: evolution of social behaviour. Oxford, UK: W. H. Freeman. (10.1093/oso/9780716745518.001.0001)

[38] Patten MM, Cowley M, Oakey RJ, Feil R. 2016 Regulatory links between imprinted genes: evolutionary predictions and consequences. Proc. Biol. Sci. **283**, 20152760. (10.1098/rspb.2015.2760) 26842569 PMC4760173

[39] Higgs MJ, Hill MJ, John RM, Isles AR. 2022 Systematic investigation of imprinted gene expression and enrichment in the mouse brain explored at single-cell resolution. BMC Genom. **23**, 754. (10.1186/s12864-022-08986-8) PMC9670596 36384442

[40] Haig D. 2003 On intrapersonal reciprocity. Evol. Hum. Behav. **24**, 418–425. (10.1016/s1090-5138(03)00063-1)

[41] Selten R. 1983 Evolutionary stability in extensive two-person games. Math. Soc. Sci. **5**, 269–363. (10.1016/0165-4896(83)90012-4)

[42] Hart S. 2002 Evolutionary dynamics and backward induction. Games Econ. Behav. **41**, 227–264. (10.1016/s0899-8256(02)00502-x)

[43] McNamara J, Houston A. 2002 Credible threats and promises. Phil. Trans. R. Soc. B **357**, 1607–1616. (10.1098/rstb.2002.1069) 12495517 PMC1693069

[44] Beviá C, Corchón L. 2024 Contests: theory and applications. Cambridge, UK: Cambridge University Press. (10.1017/9781009504409.006)

[45] Haig D. 2011 Genomic imprinting and the evolutionary psychology of human kinship. Proc. Natl Acad. Sci. USA **108**, 10878–10885. (10.1073/pnas.1100295108) 21690414 PMC3131819

[46] Haig D. 1994 Cohabitation and pregnancy-induced hypertension. Lancet **344**, 1633–1634. (10.1016/s0140-6736(94)90427-8) 7984005

[47] Robillard PY, Hulsey TC, Périanin J, Janky E, Miri EH, Papiernik E. 1994 Association of pregnancy-induced hypertension with duration of sexual cohabitation before conception. Lancet **344**, 973–975. (10.1016/s0140-6736(94)91638-1) 7934427

[48] Madrid JE, Parker KJ, Ophir AG *et al*. 2020 Variation, plasticity, and alternative mating tactics: revisiting what we know about the socially monogamous prairie vole. In Advances in the study of behavior (eds M Naguib, L Barrett, SD Healy, P Podos, LW Simmons, M Zuk), pp. 203–242, vol. 52. New York, NY: Academic Press. (10.1016/bs.asb.2020.02.001)

[49] Wilkins J, Haig D. 2002 Parental modifiers, antisense transcripts and loss of imprinting. Proc. R. Soc. B **269**, 1841–1846. (10.1098/rspb.2002.2096) PMC1691092 12350273

[50] Liu Y, Tang Y, Ye D, Ma W, Feng S, Li X, Zhou X, Chen X, Chen S. 2018 Impact of abnormal DNA methylation of imprinted loci on human spontaneous abortion. Reprod. Sci. **25**, 131–139. (10.1177/1933719117704906) 28443481

[51] Wilkins JF, Bhattacharya T. 2019 Intragenomic conflict over bet-hedging. Phil. Trans. R. Soc. B **374**, 20180142. (10.1098/rstb.2018.0142) 30966914 PMC6335451

[52] Dawkins R, Krebs JR. 1979 Arms races between and within species. Proc. R. Soc. B **205**, 489–511. (10.1098/rspb.1979.0081) 42057

[53] Scott TJ, Queller DC. 2019 Long‐term evolutionary conflict, Sisyphean arms races, and power in Fisher’s geometric model. Ecol. Evol. **9**, 11243–11253. (10.1002/ece3.5625) 31641469 PMC6802030

[54] Humphreys RK, Ruxton GD. 2020 The dicey dinner dilemma: asymmetry in predator–prey risk‐taking, a broadly applicable alternative to the life‐dinner principle. J. Evol. Biol. **33**, 377–383. (10.1111/jeb.13585) 31919916

[55] Kennedy P, Higginson A. 2025 Supplementary material from: Brinkmanship in intragenomic conflict. Figshare. (10.6084/m9.figshare.c.7737854) PMC12015577 40264365

